# Distribution of ABO Blood Group and Major Cardiovascular Risk Factors with Coronary Heart Disease

**DOI:** 10.1155/2013/782941

**Published:** 2013-08-05

**Authors:** Santanu Biswas, Pradip K. Ghoshal, Bhubaneswar Halder, Nripendranath Mandal

**Affiliations:** ^1^Division of Molecular Medicine, Bose Institute, P-1/12 CIT Scheme VIIM, Kolkata 700054, India; ^2^Department of Cardiology, N.R.S. Medical College & Hospital, 138 A.J.C. Bose Road, Kolkata 700014, India

## Abstract

The purpose of this study is to establish whether ABO blood group is related to coronary heart disease in an individual in Asian Indian Bengali population of eastern part of India. Two hundred and fifty (250) CHD patients and two hundred and fifty (250) age and sex matched healthy subjects were enrolled in the study. ABO blood group distribution in patients was compared with control group. Frequency of major cardiac risk factors was determined to find any correlation between blood groups and cardiovascular risk factors. The distribution of ABO blood groups in patients versus control group was A in 24.00 versus 21.60%, B in 30.80 versus 32.40%, O in 38.40 versus 21.60%, and AB in 6.80 versus 24.40%. The analysis showed significant difference in frequency of O (OR = 1.857, 95%CI = 1.112–3.100, *P* = 0.018) and AB (OR = 0.447, 95%CI = 0.227–0.882, *P* = 0.020) blood group between healthy controls and CHD individuals. Our results may suggest that the AB blood group decreases the risk of CHD in healthy controls, and it might be due to the higher concentration of high density lipoprotein cholesterol (HDL-c), while the O blood group increases the risk of CHD due to lower HDL-c levels in Bengali population of eastern part of India.

## 1. Introduction

Coronary heart disease (CHD) is a multifactorial disease. The etiology of CHD is complex and appears to involve interactions between genetic and environmental factors. Blood is an individual's complete and unchangeable identity. Although almost 400 blood group antigens have been reported, the ABO and Rh have been recognized as the major clinically significant blood group antigens [1]. Research on ABO group system has been of immense interest, due to its medical importance in different diseases [2–7], though the explanation for the association between ABO blood groups and some disease is still unclear. Clinical studies have shown that individuals of the A phenotype blood group are more susceptible to cardiovascular disease [8, 9]. In British men, the incidence of ischemic heart disease is higher in patients with blood group A [10]. Likewise, in the Hungarian population, again blood group A is more common in patients with CHD [11]. In a most recent study carried out in Copenhagen, Denmark, the Lewis blood phenotype Le (a-b-) was found to be associated with an increased risk for CHD in men; this appears independent of conventional cardiovascular risk factors like smoking, obesity, diabetes mellitus, high cholesterol, triglycerides, and so forth [12].

The present study was designed to explore the relation between ABO blood group and coronary heart disease in Asian Indian Bengali population of eastern part of India. We also examined the relation between ABO blood group and cardiovascular risk factors.

## 2. Methods

All subjects are Indian Bengali adults. The criteria for selecting the patients (*n* = 250) and controls (*n* = 250) have been presented in detail previously [13]. Briefly, patients having typical angina and evidence of ischemia or infarction after electrocardiographic study, tread mill test, stress echo and echocardiographic study; on the other hand, controls comprised the spouses, neighbors, and people from same work place of the patients, with the same sociocultural background, in whom the clinical history, the objective search for signals of CHD, and the electrocardiographic as well as echocardiographic examinations did not suggest the presence of that disease. All patients and controls with ancestral origin were from the Bengali eastern part of India. All patients were admitted to the hospital within 12 hours of the onset of chest pain. Blood samples for biochemical analysis were taken at the time of admission. Blood samples were collected by vein puncture into vacuum tubes containing EDTA. Any patients or controls found to have taken any lipid-lowering drugs were excluded from the study. Plasma samples were processed on the day of sample collection by centrifugation at 3000 rpm for 10 min at room temperature using tabletop centrifuge (Remi Pvt. Ltd., Mumbai, India), divided into aliquots, and stored in cryovials at −80°C until further analysis. Patients and controls were fully informed of the aim of this study. All subjects included gave their informed consent to participation in the study. The study was approved by the ethics committee of the institute involved.

Standard slide method was adopted: a drop of each of the monoclonal antisera (Anti-A, Anti-B and Anti-D) (manufactured by Agappe Diagnostics Ltd., Kerala, India) was taken on glass slides. The blood cells subjects of whose blood group is to be determined were mixed with each blood separately with the help of separate glass rods. Blood groups were determined on the basis of agglutination reaction within 5 minutes of mixing. Total cholesterol (TC) was determined enzymatically using CHOD/POD-Phosphotungstate reagent. High density lipoprotein cholesterol (HDL-c) was determined with CHOD/POD-Phosphotungstante reagent (Accurex Biomedical Pvt. Ltd., India), after precipitation with phosphotungstic acid. Triglyceride (TG) was determined using GOP-POD reagent (Accurex Biomedical Pvt. Ltd., India). Besides values of low density lipoprotein cholesterol (LDL-c) were estimated using the formulae LDL-c = TC − (HDL-c + FTG/5) [14].

After the completion of each experiment, the data were recorded on predesigned proforma and managed with Microsoft Excel software. Data entry was double checked for any human error. All calculations were performed using SPSS version 10.0 software package for Windows. Continuous variables are expressed as mean (standard deviations) and as percentages for categorical variables. Comparisons between groups were done using unpaired Student's *t*-test or chi-square (**χ** ^2^) test as appropriate. An allele frequency of the antigens was computed by application of the Hardy-Weinberg Law [15] on the basis of the number of subjects with different blood groups. ABO differences in cardiovascular risk factors were examined using post hoc analysis, after correcting the *P* value for multiple comparisons by using Bonferroni's correction. The association between different parameters and risk of CHD was examined by estimating odd ratios (ORs) with corresponding 95% confidence intervals (CIs), using univariate logistic regression analysis. Multivariate analysis was performed using multiple logistic regression (Enter method) to assess the independent adjusted relationship between different variables and CHD with independent variables being those with *P* ≤ 0.05 in univariate logistic regression analysis. The *P* value < 0.05 was considered statistically significant.

## 3. Results

During the period of this study, April 2009 to February 2011, 250 CHD patients and 250 healthy controls, participated in this study. Females constituted 18.4% of the cases and 16.8% of the controls and males constituted 81.6% of cases and 83.2% of the controls (*P* > 0.05). No significant difference was observed in mean age between cases (mean: 54.71 years) and controls (mean: 54.49 years) (*P* = 0.375).

As shown in Table 1, smoking, being an alcohol user, hypertension, family history, and increased waist circumference were highly prevalent in the group with CHD as compared to control. The mean value of TC, LDL-c, and TG was significantly higher in CHD patient compared to control; on the contrary, the mean value of HDL-c was significantly lower in patient than control. 

In Table 2 the distribution of ABO blood types in patients with CHD and controls is shown. Blood group O is more common (38.40%) in patients than controls (21.60%) (*P* < 0.0001). Blood group A is also found more often in patients (24.00%) than controls (21.60%) (*P* = 0.522). But in blood group AB, the controls are more numerous (24.40%) than patients (6.80%) (*P* < 0.0001). In blood group B also, the controls outnumber (32.40%) the patients (30.80%) (*P* = 0.271). The allele frequencies in both the patients and the controls are in order A < B < O.

We examined the contribution of major cardiovascular risk factors in subjects with ABO blood groups to all 500 patients (Table 3). We found from the analysis that HDL-c and LDL-c had significantly different mean value in the blood groups. The mean value of HDL-c was highest among AB group (36.65 mg/dL) and in O group was lowest (29.56 mg/dL) compared to other groups. But on the other hand, the LDL-c concentration was higher in O group (112.78 mg/dL) than AB group (104.80 mg/dL). In this analysis we also found that 28.7% of blood group O population had CHD in their family history. Post hoc analysis revealed significant difference of ABO blood group. In particular, compared with AB blood group, the A, B, and O groups were associated with substantial decrease of HDL-c levels. With regard to smoking and family history, differences were significant when we compared O blood group with AB blood group. There was no such significant difference of LDL-c levels found when we compared the AB blood group and O blood group but significant difference was found only when we compared the B blood group with AB blood group.

In Table 4, the univariate analysis showed significant difference in O blood group between the patient and control groups (OR = 2.263, 95% CI = 1.525–3.357. *P* < 0.0001). In addition, a significant association was found between AB blood group and CHD (OR = 0.226, 95% CI = 0.128–0.400, *P* < 0.0001). No significant difference in blood groups A and B was observed between cases and controls (*P* > 0.05); other biochemical and conventional risk factors were also significantly associated with the disease.

Multivariate analysis was performed including the variables which revealed statistically significant difference in the univariate analysis. In this analysis, LDL-c was set to be zero because it is redundant. In the end point of this analysis, AB and O blood groups along with TC, TG, HDL-c, and other conventional risk factors (i.e., hypertension, being an alcohol user, and waist circumference) remained significant, but the smoking and family history lost their significance (Table 5).

## 4. Discussions

The ABO blood group system is the most important system for blood group compatibility. However, as suggested elsewhere, ABO blood group may have additional consequences on other factors that might also contribute to the risk of thrombosis [16, 17] and deserve additional investigation particularly to explain the CHD risk. The data generated in the present study may be useful for health planners, while making efforts to face the future health challenges in the region. In short, generation of a simple database of blood groups not only provides data about the availability of human blood in case of regional calamities but also serves to enable insight into possibilities of future burden of diseases.

In the present study, we determined the frequency of ABO blood antigens in CHD patients and healthy controls. Our result showed that the AB blood group decreases the risk of CHD in healthy controls, while the O blood group is more frequent in CHD patients and increases the risk of CHD. The results obtained in this study show that, in this Bengali Asian Indian population of eastern part of India, the prevalence of CHD in blood group O is invariably higher than in all other ABO blood groups, but Whincup et al. [10] from England and from other parts of Europe [18, 19] or USA [12], found that frequency of A blood group was more than any other ABO blood groups in CHD patients.

In the analyses of the relation between the ABO blood group and major cardiovascular risk factors the only association of note was that O blood group, probably by association with lower HDL-c levels, smoking habit, and family history, significantly increases the risk of CHD, and contributes substantially to the incidence of CHD in the studied populations. In the two previous reports [20, 21], only association was found between blood group A and serum total cholesterol concentration among the major cardiovascular risk factors. The higher concentrations of HDL-c in subjects of blood group AB seemed to contribute to the protective role of CHD events in subjects of controls group.

The limitation of the present study is the lack of follow-up data, mostly due to the lack of patient compliance. We have also a limitation in our study regarding the estimation of the extent and burden of atherosclerosis by doing coronary angiography and multidetector row computed tomography.

## 5. Conclusion

The racial and ethnic distribution of blood groups and size of sample are important factors for predicting the CHD risk. Blood type needs to be considered together with other risk factors to understand the individual patient's risk. The identification of genetic and environmental factors among racial and ethnic groups should offer some insights into the observed epidemiological data and advance opportunities to better understand the control and development of CHD.

## Figures and Tables

**Table 1:** Characteristic of Asian Indian Bengalee population in eastern part of India in the case and control groups.

Variable	Case (*N* = 250)	Control (*N* = 250)	*P* value
No. (%) of smokers	150 (60%)	70 (28%)	<0.0001
No. (%) of alcohol users	61 (24.4%)	35 (14%)	0.003
No. (%) of cases with hypertension	119 (47.6%)	84 (33.6%)	0.001
No. (%) of cases with family history	81 (32.4%)	38 (15.2%)	<0.0001
WC (cm)	87.24 (6.37)	85.28 (5.30)	<0.0001
TC (mg/dL)	191.11 (40.37)	168.51 (42.83)	<0.0001
TG (mg/dL)	183.09 (55.16)	141.47 (57.75)	<0.0001
HDL-c (mg/dL)	28.81 (7.28)	34.60 (8.52)	<0.0001
LDL-c (mg/dL)	125.49 (37.60)	105.62 (37.89)	<0.0001

Continuos variable are expressed as mean (SD) and compared by Student's *t*-test. Categorical variables were expressed as percentage (%) and compared by chi-square test.

TC: total cholesterol, SD: standard deviation, TG: triglycerides, HDL-c: high density lipoprotein cholesterol, LDL-c: low density lipoprotein cholesterol, WC: waist circumference.

**Table 2:** Distribution of the ABO blood types in patients with CHD and controls.

Sample	*n*		Phenotype frequency				Allele frequency		
			A	B	O	AB	A	B	O
Patients	250	Abs. no.	60	77	96	17	0.193	0.238	0.567
		%	24.00	30.80	38.40	6.80			
Controls	250	Abs. no.	54	81	54	61	0.265	0.337	0.398
		%	21.60	32.40	21.60	24.40			

**Table 3:** Distribution of major cardiovascular risk factors in all samples (*n* = 500) in accordance with ABO blood groups.

Variables (mean (SD))	Blood group				Test for heterogeneity^1^
	AB (*n* = 78)	A (*n* = 114)	B (*n* = 158)	O (*n* = 150)	
TC (mg/dL)	174.08 (44.52)^2^	181.24 (36.29)	185.28 (49.77)	174.96 (38.43)	*P* = 0.117
TG (mg/dL)	163.11 (39.02)	159.24 (64.28)	164.01 (66.02)	161.38 (59.03)	*P* = 0.928
HDL-c (mg/dL)	36.65 (12.04)	30.81 (5.78)^5^	32.13 (7.39)^5^	29.56 (8.11)^5^	*P* < 0.0001
LDL-c (mg/dL)	104.80 (40.71)	118.58 (32.29)	120.34 (44.05)^4^	112.78 (35.79)	*P* = 0.025
WC (cm)	85.55 (4.03)	86.62 (6.93)	86.04 (6.08)	86.56 (5.72)	*P* = 0.557
Smoker	25 (32.1%)^3^	41 (36%)	78 (49.4%)	78 (52%)^4^	*P* = 0.007
Alcohol user	9 (11.5%)	40 (35.1%)^5^	36 (22.8%)	14 (9.3%)	*P* < 0.0001
Hypertension	25 (32.1%)	46 (40.9%)	66 (41.8%)	67 (44.7%)	*P* = 0.457
Family history	8 (10.3%)	27 (23.7%)	41 (25.9%)	43 (28.7%)^4^	*P* = 0.037

^1^Derived from analysis of covariance that evaluated the associations between major cardiovascular risk factors (dependent factor) and blood group (independent factor), after adjustment for age (in years) and sex.

^2^Mean (SD) (all such values).

^3^Number (percentage in individual group) (all such values).

^4,5^Significantly different from AB blood group (ANOVA with Bonferroni's correction): ^4^ *P* < 0.05, ^5^ *P* < 0.01.

A indicates A blood group; B indicates B blood group; O indicates O blood group; AB indicates AB blood group; TC: total cholesterol; TG: triglycerides; HDL-c: high density lipoprotein cholesterol; LDL-c: low density lipoprotein cholesterol; WC: waist circumference.

**Table 4:** Risk of coronary heart disease (CHD) by ABO blood group and other risk factors in the study patients as compared to study controls.

Variables	Odd ratio	95% CI	*P* value
A	1.146	0.754–1.742	0.523
B	0.834	0.574–1.212	0.341
O	2.263	1.525–3.357	0.000
AB	0.226	0.128–0.400	0.000
TC	0.987	0.982–0.991	0.000
TG	0.986	0.983–0.990	0.000
HDL-c	1.102	1.073–1.132	0.000
LDL-c	0.986	0.981–0.991	0.000
Smoker	1.851	1.294–2.647	0.001
Hypertension	1.795	1.251–2.576	0.001
Alcohol user	1.983	1.253–3.138	0.003
WC	0.945	0.916–0.974	0.000
Family history	2.674	1.730–4.132	0.000

Odd ratio (OR) calculated by univariate logistic regression analysis. A indicates A blood group; B indicates B blood group; O indicates O blood group; AB indicate AB blood group; TC: total cholesterol; TG: triglycerides; HDL-c: high density lipoprotein cholesterol; LDL-c: low density lipoprotein cholesterol; WC: waist circumference.

**Table 5:** Multivariate logistic regression analysis of different variables of coronary heart disease among Asian Indian Bengalee population in the eastern part of India.

Variables	Odd ratio	95% CI	*P* value
TC	0.990	0.984–0.996	0.001
TG	0.990	0.985–0.994	<0.0001
HDL-c	1.097	1.063–1.132	<0.0001
AB	0.447	0.227–0.882	0.020
O	1.857	1.112–3.100	0.018
Smoker	1.340	0.897–2.003	0.153
Hypertension	1.772	1.130–2.779	0.013
Alcohol user	2.004	1.100–3.650	0.023
WC	0.917	0.882–0.954	<0.0001
Family history	1.430	0.821–2.490	0.206

Odd ratio (OR) calculated by multiple logistic regression analysis. O indicates O blood group, AB indicates AB blood group; TC: total cholesterol; TG: triglycerides; HDL-c: high density lipoprotein cholesterol; WC: waist circumference.
